# Chryseobacterium indologenes Ventilator-Associated Pneumonia in an Elderly Patient: A Case Report

**DOI:** 10.7759/cureus.27426

**Published:** 2022-07-28

**Authors:** Mahdi M Fadlallah, Darine M Kharroubi, Zeinab Zeineddine, Sarah M Salman

**Affiliations:** 1 Department of Laboratory Medicine, Al-Zahraa Hospital University Medical Center, Beirut, LBN; 2 Department of Laboratory Medicine, Lebanese University, Faculty of Medical Sciences, Beirut, LBN

**Keywords:** multidrug-resistant bacteria, tracheal aspirate, ventilator-associated pneumonia, elderly, healthcare-associated infection, c. indologenes

## Abstract

*Chryseobacterium indologenes* is a rare non-fermenting gram-negative pathogen that can cause opportunistic infections in humans. Most infections are nosocomial and acquired through contaminated devices such as ventilators, endotracheal tubes, and indwelling catheters. An increasing number of infections have been reported in recent years after the first reported case of ventilator-associated pneumonia in 1993. Blood, lung, ocular, and peritoneal infections, among others, have been reported. The high rate of intrinsic resistance to broad-spectrum antibiotics is a matter of concern since there are no standard guidelines for the management and treatment of this infection. Here, we present the case of a 94-year-old female who was admitted and intubated after a cerebral vascular accident. During her stay, she developed a fever. The deep tracheal aspirate culture was positive for gram-negative bacilli with smooth, circular, and yellow-pigmented colonies that were later identified as *C. indologenes*. Antimicrobial susceptibility tests done with VITEK 2 and by the Kirby-Bauer disc diffusion method showed susceptibility to ciprofloxacin, minocycline, and trimethoprim-sulfamethoxazole and resistance to all other tested antimicrobials. The infection was successfully treated with ciprofloxacin antibiotic. To the best of our knowledge, this is the first reported case of *C. indologenes* infection in Lebanon.

## Introduction

*Chryseobacterium indologenes* (*C. indologene*s) (known in the past as *Flavobacterium indologenes*) belong to the *Flavobacterium* species CDC group IIb [1]. It is a non-motile, oxidase-positive, catalase-positive, non-fermenting gram-negative pathogen that can cause opportunistic infections in humans [2,3]. Although ubiquitous in nature, this organism is a rare pathogen and normally non-existing in the human microflora [1,4]. Mostly, *C. Indologenes* infections are nosocomial [5].

Despite *C. indologenes* having low virulence, it can be a life-threatening pathogen causing significant morbidity and mortality among patients with predisposing conditions such as extremes of age, prolonged antibiotic therapy, recent surgery, immunodeficiency, malignancies, presence of invasive devices, and indwelling catheters [6]. Choice of antibiotic therapy for the treatment of *C. indologenes* infections is difficult due to the unpredictability and the rapid evolution of its antimicrobial resistance. It is often resistant to many antimicrobial agents used for the empiric treatment of gram-negative infections [4]. In the present manuscript, we report a rare case of *C. indologenes* pneumonia in an elderly patient on mechanical ventilation.

## Case presentation

A 94-year-old female known to have hypertension and coronary artery disease presented to the emergency department with symptoms of decreased level of consciousness and right-sided weakness. Clinical examination on arrival at our facility showed raised blood pressure (170/80 mmHg), tachycardia (143/min), normal body temperature (36.8℃), oxygen saturation (SpO_2_) of 96%, and Glasgow Coma Scale of 7. Consequently, emergent intubation of the patient was performed. A neurological exam revealed a neurological deficit in terms of weakness in the right arm and leg. MRI results showed acute cerebral ischemia in the left middle cerebral artery territory. Laboratory tests revealed polymorphonuclear leukocytosis (White blood count: 19.6×10^3^/µL, neutrophils: 18×10^3^/µL), normal hemoglobin (13 g/dL), and platelet count (184×10^3^/µL), raised acute phase reactants (C-reactive protein 397 mg/L), and numerous leukocytes in urine (Table 1). Urine culture was taken and broad-spectrum antibiotics, ceftriaxone 2 g intravenous (IV) once daily, and piperacillin/tazobactam 4.5 g IV every 6 hours were started. The patient was admitted to the intensive care unit (ICU) for management of her cerebrospinal vascular insult. Urine culture was positive after 48 hours for *Escherichia coli *(>10^5^ CFU/mL) which was sensitive to both antibiotics already started on the day of admission.

**Table 1:** Laboratory Results on Admission RBC: red blood cells; WBC: white blood cells; HPF: high-power field; CFU: colony forming unit; PT: prothrombin time; INR: international normalized ratio; PTT: partial thromboplastin time; BUN: blood urea nitrogen; CRP: C-reactive protein; ALT: alanine aminotransferase; GGT: gamma-glutamyl transferase; hs: high sensitive; SG: specific gravity; pH: potential of hydrogen

Investigation	Patient value	Unit	Reference value
Hematology			
RBC	4.19	10^6^/µL	4.0-5.5
Hemoglobin	13.00	g/dL	12-16
Hematocrit	38.6	%	37-46
WBC	19.6	10^3^/µL	4.0-11.0
Neutrophils	18	10^3^/µL	1.6-7.2
Lymphocytes	1.23	10^3^/µL	0.95-3.07
Monocytes	0.43	10^3^/µL	0.26-0.81
Platelets	184	10^3^/µL	150-400
Coagulation			
PT	14.7	Seconds	10.5-13.0
INR	1.3	-	0.85-1.2
PTT	36.8	Seconds	27-39
Chemistry			
BUN	34	mg/dL	6-20
Creatinine	0.85	mg/dL	0.51-0.95
Sodium	141	mmol/L	136-145
Potassium	3.52	mmol/L	3.7-5.3
Chloride	103.6	mmol/L	98-107
Bicarbonate	17.2	mmol/L	23-29
Calcium	8.29	mg/dL	8.5-10.5
Phosphorus	3.8	mg/dL	2.7-4.8
Magnesium	2.05	mg/dL	1.7-2.6
CRP	397	mg/L	0-5
ALT	18.1	U/L	5-41
Alkaline phosphatase	59	U/L	35-129
GGT	13	U/L	6-42
Total protein	57.7	g/L	60-83
Albumin	18	g/L	36-53
Globulin	39.7	g/L	21-34
Troponin I hs	0.038	ng/mL	<0.015
Urine Test			
SG	1.015	-	1.005-1.030
pH	6		4.5-8
Nitrite	Negative	-	Negative
Protein	Negative	-	Negative
Glucose	Negative	-	Negative
Leukocyte esterase	3+	-	Negative
Hemoglobin	1+	-	Negative
WBC	Numerous	/HPF	0-5
RBC	2-4	/HPF	0-5
Gram stain	Gram-negative bacilli	-	-
Culture	Escherichia coli >10^5^	CFU/mL	No growth

**Figure 1:**
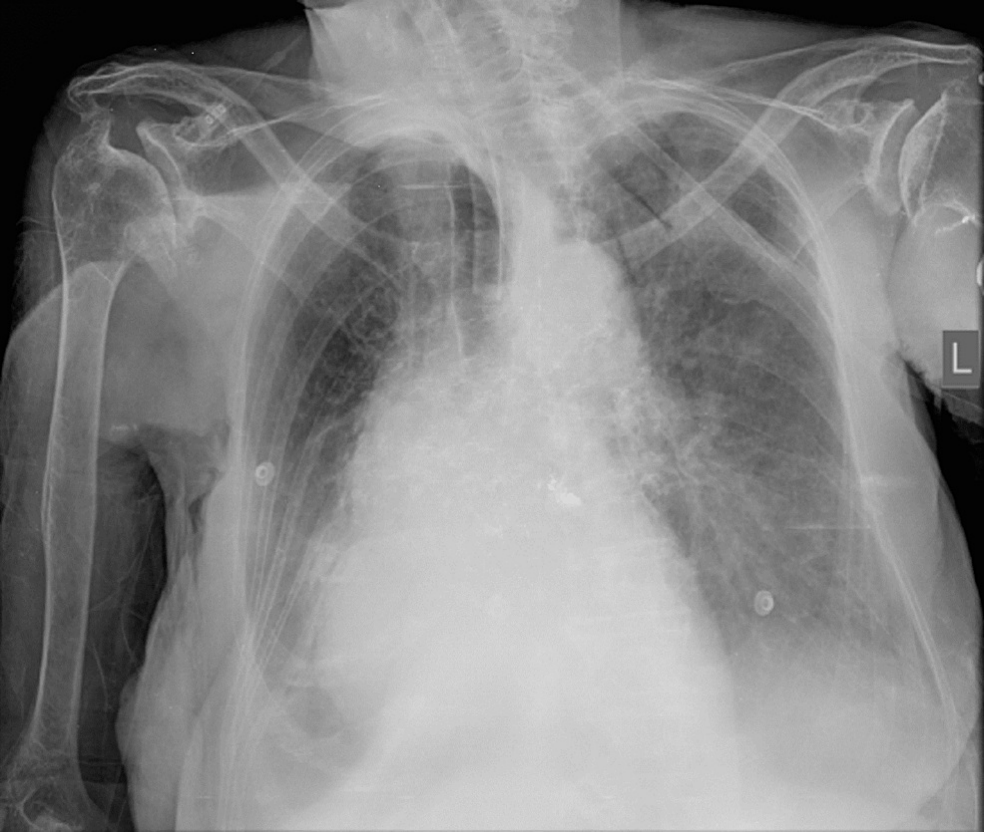
Chest X-ray Asymmetrical interstitial infiltrates in both lung fields

**Figure 2:**
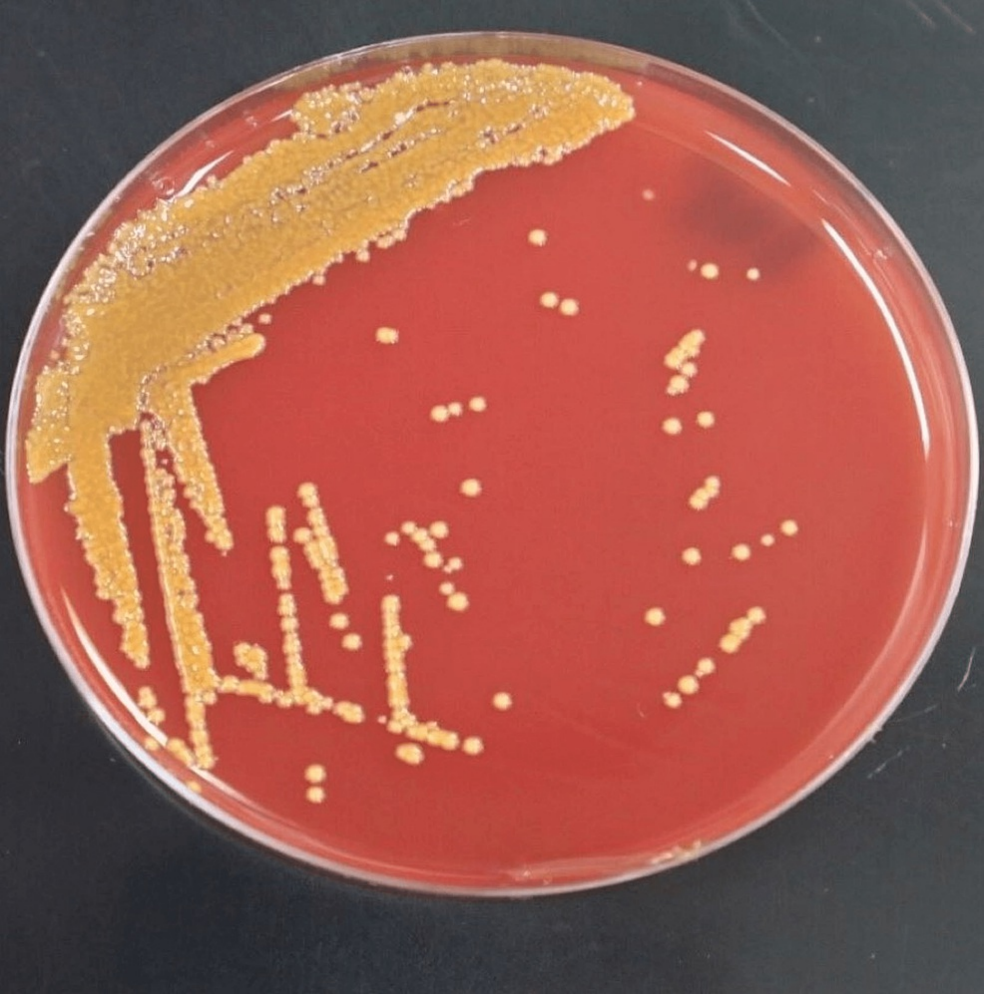
Subculture of Isolated Chryseobacterium indologenes on 5% Sheep Blood Agar This figure shows yellow-pigmented, smooth, and circular bacterial colonies of *C. Indologenes* isolated on 5% Sheep Blood Agar Plate

After 4 days of stay in the ICU, the patient developed a fever spike of 39℃. The remaining vital signs were stable. Blood, urine, and deep tracheal aspiration cultures were taken. Chest radiograph revealed an asymmetrical interstitial infiltrate in both lung fields (Figure 1). Blood and urine cultures were negative. However, deep tracheal aspirate was positive for gram-negative bacilli. On 5% sheep blood agar, the colonies were smooth, circular, and yellow-pigmented (Figure 2). No growth was observed on MacConkey agar. Catalase, oxidase, and indole tests were positive. The urease test was negative. A color change of colonies from yellow to red was observed after the addition of 10% KOH solution.

VITEK 2 system (BioMérieux, France) was used for the final identification and sensitivity of the organism. *C. indologenes* was identified. Environmental cultures taken from the ICU room were negative. Antimicrobial susceptibility was determined by VITEK 2 and by the Kirby-Bauer disc diffusion method as per clinical and laboratory standards institute (CLSI) protocols (Table 2). The yellow pigmentation was also observed on Mueller Hinton agar used for the Kirby-Bauer disc diffusion method.

**Table 2:** Chryseobacterium indologenes Minimum Inhibitory Concentration Values for Various Antimicrobial Agents MIC: minimum inhibitory concentration (µg/ml); R: resistant; I: intermediate; S: susceptible; TMP/SMZ: trimethoprim/sulfamethoxazole

Antibiotic	MIC	Interpretation
Piperacillin/tazobactam	≥ 128	R
Ticarcillin/clavulanic acid	≥ 128	R
Ceftazidime	≥ 64	R
Cefepime	≥ 64	R
Cefotaxime	≥ 64	R
Aztreonam	≥ 64	R
Meropenem	≥ 16	R
Imipenem	≥ 16	R
Ciprofloxacin	= 1	S
Levofloxacin	= 4	I
Amikacin	≥ 16	R
Gentamicin	≥ 16	R
Tobramycin	≥ 16	R
Minocycline	≤ 0.5	S
Tetracyclin	= 8	I
TMP/SMZ	≤ 20	S
Colistin	≥ 16	R

Ciprofloxacin (400 mg IV every 12 hours) was started. Two days later, the fever stopped. Repeated deep tracheal aspirate culture was negative. The patient was then extubated and discharged home on conservative treatment for her cerebral ischemia.

## Discussion

Genus *Chryseobacterium*, belonging to the family *Flavobacteriaceae*, is ubiquitous in the environment and is found naturally in soil and humid surfaces [3,7]. As the organism can survive chlorination, it has the potential to colonize water supplies and become a potential reservoir for infection. Thus, contaminated devices and tubes containing fluids such as respirators, endotracheal tubes, indwelling catheters, feeding tubes, and syringes are likely to be potential sources of infection [1,8]. Since the firstly reported infection in 1993 of ventilator-associated pneumonia caused by *C. indologenes*, an increasing number of infections have been described in recent years [2,3]. Among these cases, pneumonia, bacteremia, endocarditis, ocular infections, cellulitis, pleural, and peritoneal infections have been recognized [6,7,9-13]. Infections of the urinary tract, biliary tract, lumboperitoneal shunt, burn wound, and surgical site have been also reported [7,14]. It is an opportunistic pathogen, where extremes of age and immunosuppression are among the most common risk factors [15]. Therefore, it is a nosocomial infection, where the majority of cases were reported in hospitalized patients; undergoing invasive procedures, receiving respiratory assistance, mechanical ventilation, and a long period of broad-spectrum antibiotic treatment. Moreover, small outbreaks of lower respiratory tract infection in ICU elderly patients were detected. Hence, once isolated, the implementation of strict infection control measures for this organism in the hospital is warranted to limit its spread [15,16].

On the other hand, in media cultured with *C. indologenes*, it is easily recognized due to the production of flexirubin, a clear yellow to orange pigment on the blood agar plate within 24 hours of incubation [1,16]. Bacterial identification methods include manual biochemical tests together with Phoenix (Becton Dickinson (BD), San Diego, CA, USA) and VITEK 2 (bioMérieux, Marcy L'Etoile, France) automated systems and matrix-assisted desorption ionization-time of flight mass spectrometry (MALDI-TOF MS) which is broadly applied in the identification of non-fermenting gram-negative rods [17,18]. However, VITEK 2 automated system was used in our case, which has shown a similar capability in *C. indologenes *detection in comparison to MALDI-TOF MS [18].

Furthermore, biofilm and protease production by *C. indologenes* on indwelling devices and foreign materials play a potential virulence factor in invasive infections [3,5]. These biofilms were highly detected in respiratory isolates (sputum) in comparison to other sites [18].

Antimicrobial susceptibility is a matter of concern due to the high rate of intrinsic resistance to broad-spectrum antibiotics and the lack of standard guidelines for the management and treatment of this infection. More importantly, *C. indologenes* are well known resistant to aminoglycosides, clindamycin, chloramphenicol, erythromycin, tetracycline, teicoplanin, and colistin. The production of molecular class A beta-lactamase and class B carbapenem hydrolyzing beta-lactamase confer an intrinsic resistance to cephalosporins and carbapenems, respectively [1,3,15]. As per the SENTRY antimicrobial surveillance program (1997-2004), antimicrobials that showed high activity against *Chryseobacterium* species include quinolones, trimethoprim-sulfamethoxazole (TMP/SMZ), and piperacillin/tazobactam, in addition to piperacillin, cefepime, and rifampin [15]. Despite the lower susceptibility to ciprofloxacin in comparison to newer quinolones (garenoxacin, gatifloxacin, and levofloxacin), it was described as an effective treatment for *C. indologenes* infection [1,15]. In our case, *C. indologenes *isolates were susceptible to ciprofloxacin, administered to the patient and resulted in the eradication of the infection. However, an evolving pattern of resistance over time was hypothesized where a more recent report conducted by Chen et al. in 2013, showed decreased susceptibility to quinolones and piperacillin/tazobactam [3].

## Conclusions

To the best of our knowledge, this is the first documented case report of *C. indologenes *ventilator-associated pneumonia in Lebanon. This report promotes awareness about *C. indologenes* as an emerging, opportunistic, multidrug-resistant, and potential pathogen, especially in immunocompromised and hospitalized patients. Rapid and accurate identification and susceptibility testing of *C. Indologenes* is essential in treating patients and preventing complications. However, the various and high-resistance antimicrobial susceptibility patterns of this microorganism, in addition to the absence of standard guidelines for treatment may emphasize an individualized treatment. Isolation of this pathogen requires strict infection control measures to prevent outbreaks in hospitalized patients.
